# Induced gamma band activity from EEG as a possible index of training-related brain plasticity in motor tasks

**DOI:** 10.1371/journal.pone.0186008

**Published:** 2017-10-05

**Authors:** Carlos Amo, Luis De Santiago, Daniel Zarza Luciáñez, José Miguel León Alonso-Cortés, Miguel Alonso-Alonso, Rafael Barea, Luciano Boquete

**Affiliations:** 1 Department of Electronics, Universidad de Alcalá, Alcalá de Henares, Spain; 2 Research Department—Clinical Neurophysiology, Ecnis-Sigma SL, Madrid, Spain; 3 Department of Clinical Neurophysiology, Hospital Nisa Pardo de Aravaca, Madrid, Spain; 4 Department of Clinical Neurophysiology, Complejo Asistencial Universitario de Palencia, Hospital Río Carrión, Palencia, Spain; 5 Laboratory of Bariatric and Nutritional Neuroscience, Center for the Study of Nutrition Medicine, Beth Israel Deaconess Medical Center, Harvard Medical School Boston, MA, United States of America; Nanjing Normal University, CHINA

## Abstract

The aim of this study was proposing gamma band activity (GBA) as an index of training-related brain plasticity in the motor cortex. Sixteen controls underwent an experimental session where electroencephalography (EEG) activity was recorded at baseline (resting) and during a motor task (hand movements). GBA was obtained from the EEG data at baseline and during the task. Index of plasticity (IP) was defined as the relationship between GBA at the end of the motor task (GBA_M_FIN_), divided by GBA at the beginning of the task (GBA_M_INI_) for movements of both hands. There was a significant increase in GBA at the end of the task, compared to the initial GBA for the motor task (GBA_M_FIN_ > GBA_M_INI_). No differences were found at baseline (GBA_B_FIN_ ≈ GBA_B_INI_). Individual IP values had a positive (r = 0.624) and significant correlation with subject’s handedness. Due to plastic changes, GBA could indirectly but objectively reveal changes in cerebral activity related to physical training. This method could be used as a future diagnostic test in the follow-up of patients undergoing rehabilitation. It could also have potential applications in the fields of sports medicine.

## Introduction

Plasticity is an intrinsic property of the human brain that enables it to adapt to variations in the physical environment, physiologic changes and new experiences. Plastic changes occur by modifying pre-existing neuronal connections through changes in cortico-cortical and cortico-sub-cortical networks in response to new afferent impulses or efferent demands. This way, the initial modifications changed-mediated at molecular and cellular level can be followed by the establishment of new connections through dendritic growth and arborisation [1].

One of the founding fathers in the study of brain plasticity, Santiago Ramón y Cajal, already argued in his book, *Textura del Sistema Nervioso* [2], that the ability to modify behavior must have an anatomical basis in the brain and thus, he extended the notion of plasticity to a neural substrate. In this book he proposed, as an example, the skills of a pianist which require many years of mental and physical practice.

With the acquisition of new abilities, the brain changes initially through the reinforcement of the established neuronal networks and, subsequently, with the generation of new circuits. These plastic changes imply an increase in the activated cortical areas. The phenomenon is well illustrated in the following motor experiment [3]. During five days of practice, a group of healthy volunteers were trained to play a specific musical sequence with the five fingers of one hand. With this training it was possible to significantly improve the ability to perform the sequence and decrease the number of errors. Motor cortical areas were mapped through focal transcranial magnetic stimulation (TMS) that can be used to treat and measure the nervous system [4,5]. This technique confirms a significant increase of the cortical representation of the extensor and flexor muscles of the fingers of the used hand. The same results were obtained with the mental practice of the same motor sequence of the fingers (imagined movement).

Physiological changes produced by plasticity can be long-lasting. For example, it has been confirmed that after several years of training, the representation of the motor cortical areas is very different for the fingers of the right and left hand of professional violinists [6]. It is also observed in sports practice that physical training causes plastic modulation of neuronal circuits [7].

An objective way of quantifying plastic changes due to motor training could be measuring the activity of cortical networks involved during the voluntary movement. This variation of the cortical activity has been described in several experiments with electroencephalography (EEG) as an event-related synchronization (ERS) in the frequencies of the gamma band (>30 Hz) [8–10].

Gamma band activity (GBA) is widely distributed in all cerebral structures, as well as in the retina and olfactory tract. It is generated by GABA, glutamate and acetylcholine neurotransmitters and has been linked to key brain functions such as perception, attention, memory, consciousness, synaptic plasticity and motor control [11].

The aim of this study is to describe a new EEG-based method to quantify the effects of motor training by measuring plastic changes in the brain cortex, specifically via GBA related to motor activity. The measure of these plastic changes could serve as a quantitative biomarker to motor cerebral disorders and for evaluation in rehabilitation therapies or in sport medicine.

As repeated activity of a specific movement is known to lead to new interconnections, implying a higher number or neurons [1], we hypothesized that:

1. Due to plastic changes, the power of GBA would be significantly higher at the end of a motor task (more neurons involved in the movement) than at the beginning (fewer neurons involved in the movement).
2. An index of plasticity (IP) would best represent these changes. This index would be defined as the ratio between the GBA at the end of a motor task and the GBA at the beginning (IP = GBA_M_FIN_ / GBA_M_INI_).

## Material and methods

This study was approved by the Ethics Committee of the University of Alcalá (Madrid, Spain). Informed written consent was obtained from all participants prior to any study procedure.

### Sample

A total of 16 subjects took part in the experiment (6 females, 10 males) with a mean age of 27.12 years (range: 20–47). All subjects were healthy, with no previous known medical, psychiatric or neurological disease, including head injury, epilepsy, past history of alcoholism or drug abuse. Only medication-free subjects were allowed to take part in the study.

### Experimental procedures

Participants sat on a comfortable chair facing a computer monitor placed 0.8 m away from their head. Forearms were in resting position on a table and with hand palms facing down.

The experiment consisted of two parts (baseline and motor experiment), conducted in a single session for each subject.

During the baseline part of the experiment, subjects remained with eyes open staring at a black dot displayed at the center of a white screen. A total of 18 minutes of baseline activity were acquired, divided in 3 parts (6 minutes) with a resting period between them of approximately one minute. The purpose of this first part was to obtain baseline GBA (spontaneous gamma oscillations) of the EEG.

In the second part (motor experiment), participants performed a motor task immediately after a signal (cue) was displayed on the screen. The task consisted in a quick extension of the wrist followed by a light relaxation. Subjects practiced this exercise previously in a training session (repeating 10 times the motor task with each hand: the cue was displayed on the screen and the subject moved the hand). The purpose of this second part was to obtain the movement-related GBA (induced gamma oscillations) of the EEG.

The motor task was performed in trials of two seconds that started at t = 0 seconds with the cue being displayed at the center of the screen for 150 ms, followed by a white screen which remained until the beginning of the next trial (t = 2 s).

The complete motor experiment consisted of 500 trials (500 extensions of the wrist) for each hand divided in 5 runs. Each run consists of 100 consecutive trials. Right and left hand runs were alternated in order to prevent from muscle fatigue. The total duration of the motor experiment was approximately 40 minutes.

Throughout the experiment (baseline and motor), subjects remained starring at a fixed dot on the screen to control for variability in eye movements and they were instructed to avoid blinking, swallowing or any other movements apart from the one required with the hand.

At the end of the experimental sessions participants were asked to fill in the Edinburgh Handedness Inventory scale (EHI), to determine manual laterality [12]. The final sample was composed by 11 right-handed, 2 left-handed and 3 ambidextrous subjects.

### Data acquisition

EEG was acquired with a 32-channel Micromed system, model Handy EEG SD32 and the acquisition software *System Plus Evolution* (Micromed SpA, Treviso, Italy). We used an A/D sigma-delta converter with 22 bits of resolution, sampling frequency 2048 Hz, band pass filters 0.15–537.53 Hz, a notch filter of 50 Hz and electrode impedances < 10 kΩ.

We used three EEG channels (Cz, FPz and Pz), two channels of Electrooculogram (EOG) and four channels of Electromyogram (EMG). EEG was recorded using Ag/AgCl electrodes located according to the 10/20 system. Cz was the active electrode; FPz and Pz were reference and ground electrodes respectively. EOG was recorded to monitor the ocular vertical-horizontal movements with an electrode placed over the external ocular edge of the right eye and the other one placed under the external ocular edge of the left eye. EMG was obtained through two surface electrodes (active plus reference) placed above each of the forearms over the extensor *carpi radialis longus* muscle.

During recordings the lights of the laboratory were turned off and we used batteries for the acquisition equipment and for the computer with the purpose of reducing as much as possible the induction of the alternating signal to 50 Hz in the wires of EEG. A more detailed description of the experimental methodology and procedures can be found in a recent publication [10].

### Data analysis

The EEG, EMG and EOG signals were analyzed offline using *Matlab 2009b* (The Math Works Inc. Natick, MA, USA) and *FieldTrip* [13] and the data were processed in European Data Format.

The signal of the baseline part of the experiment (acquired without triggers) was divided into segments of the same duration as for the trials of the motor experiment (2 seconds) in order to compare them. The signal of the motor experiment was divided according to the 2 seconds trials based on the triggers corresponding to each cue.

EEG signal was high-pass filtered (1 Hz) to remove baseline drift and low-pass filtered (100 Hz). In addition, a 49-51Hz notch filter has been implemented to minimize power line interference. Butterworth method (fourth-order, zero phase shift) has been used to implement the filters.

Using the *ft_RejectArtifact* and the *ft_RejectVisual* functions of FieldTrip, EMG and EOG artifacts were discarded applying the function’s standard parameters and the corresponding EMG (30–100 Hz) and EOG (1–70 Hz) filters. The linear trend error was eliminated using the *ft_Detrend* function.

After these processing steps, we obtained the following parameters from EMG and EEG (free of artifacts), average value for each subject and for all of them (grand average).

#### EMG parameters

Mean amplitude (EMG_MEAN_) and maximum amplitude (EMG_MAX_) were calculated in each trial. Also the peak-average ratio (EMG_PAR_) was defined as: ${EMG}_{PAR}=\frac{{EMG}_{MAX}}{{EMG}_{MEAN}}$.

#### Calculation of GBA

The GBA was obtained from the spectral power values for the frequency band 30–60 Hz and was calculated with multi taper Fast Fourier Transform (FFT), using the *ft_freqanalysis* function. Results were expressed as the average value of the power spectral density (PSD) in μV^2^.

For each subject, we calculated the PSD separately for each trial and the average of all trials of the baseline and motor task.

Based on the average values of the PSD, the following parameters were defined:

- Baseline GBA (GBA_B_): the PSD during the baseline experiment, representing the spontaneous gamma oscillations.
- Motor GBA (GBA_M_): the PSD during the motor task of the right (GBA_MR_) or left (GBA_ML_) hand, representing the induced gamma oscillations.

#### Calculation of the ERS (event related synchronization) from GBA

ERS was defined by normalizing the GBA_M_ values with respect to GBA_B_:

- ERS during motor activity of the right hand: ${ERS}_{R}=\frac{{GBA}_{MR}}{{GBA}_{B}}$
- ERS during the motor activity of the left hand: ${ERS}_{L}=\frac{{GBA}_{ML}}{{GBA}_{B}}$

#### Calculation of the motor task intervals

With the purpose of comparing the GBA at the beginning and end of the motor task, three groups of trials (free of artifacts) were defined:

- initial [GBA_M_INI_ = 100 first trials of the motor task],
- middle [GBA_M_MID_ = 100 trials in the middle of the motor task],
- final [GBA_M_FIN_ = 100 last trials of the motor task].

The different groups of GBA_M_ are identified with letters “R” for the right hand (GBA_MR_INI_, GBA_MR_MID_, GBA_MR_FIN_) and “L” for the left one (GBA_ML_INI_…).

#### Calculation of the index of plasticity (IP)

Table 1 shows the IP definitions for basal and motor parameters.

**Table 1:** IP definitions for each case.

Basal	Motor		
Increase of GBA_B_	Right IP	Left IP	IP difference between sides
${∆GBA}_{B}=\frac{{GBA}_{B\_FIN}}{{GBA}_{B\_INI}}$	${IP}_{R}=\frac{{GBA}_{MR\_FIN}}{{GBA}_{MR\_INI}}$	${IP}_{L}=\frac{{GBA}_{ML\_FIN}}{{GBA}_{ML\_INI}}$	IP_R_−IP_L_

The variation of basal activity cannot be considered as a plastic change so is not defined as IP. The variation or increase of the basal activity is defined as ΔGBA_B_ and does not imply functional changes. In order to calculate ΔGBA_B_ (Table 1), the average values of the initial and final periods of the baseline experiment (GBA_B_INI_ and GBA_B_FIN_) are obtained. These initial and final periods of the baseline experiment are equivalent and can be compared to the ones in the motor task.

**Fig 1:**
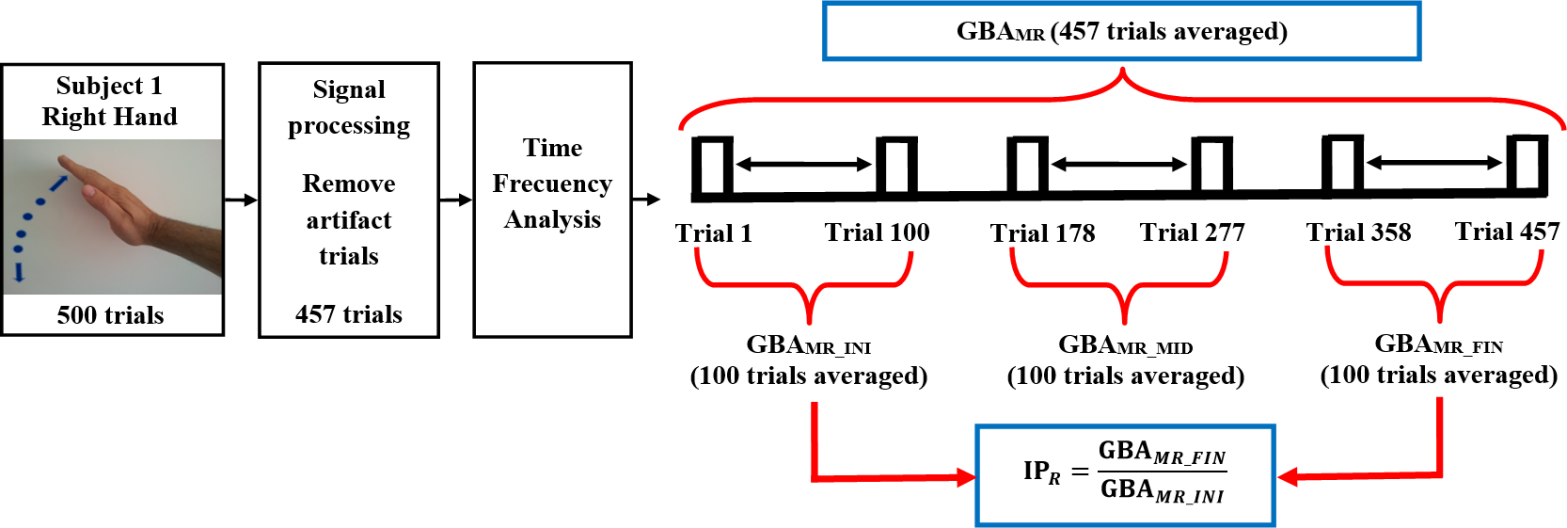
Example of the procedure used for the calculation of IP_R_ (subject 1, right hand). In this example 457 of the 500 trials of the motor task were obtained free of artifact. The GBA_MR_ value expressed as μV^2^ is obtained through the time-frequency analysis of the valid trials (457). For the calculation of the IP_R_, the average value of the last 100 valid trials is obtained (GBA_MR_FIN_) and it is divided by the average value of the first 100 valid trials (GBA_MR_INI_). The IP has no units. GBA = Gamma band activity, IP = Index of plasticity, MR = Motor Right, INI = Initial, MID = Middle, FIN = Final.

IP was defined as the relationship between the GBA at the end of the motor task (GBA_M_FIN_) divided by the GBA at the beginning of the same motor task (GBA_M_INI_) for movements of both hands. The IP index difference was defined with the purpose of verifying if there were differences according to the manual laterality of each subject. Fig 1 shows the procedures to calculate the IP_R_ (which is the same for the IP_L_ and for ΔGBA_B_).

### Statistical analysis

All statistical analyses were performed using the SPSS 21.0 statistical package software (SPSS Inc. Chicago, Illinois, USA). Student’s t test (t test) was used to compare the averages and Pearson’s linear correlation was used to compare quantitative variables. The Gaussian distribution assumption was tested using the Kolmogorov–Smirnov test.

The results are expressed as average and confidence interval (CI 95%). The significance value for the differences was set at p < 0.05.

## Results

We present the results based on the steps taken to test the study hypotheses.

### First step: Obtain the GBA values

**Fig 2:**
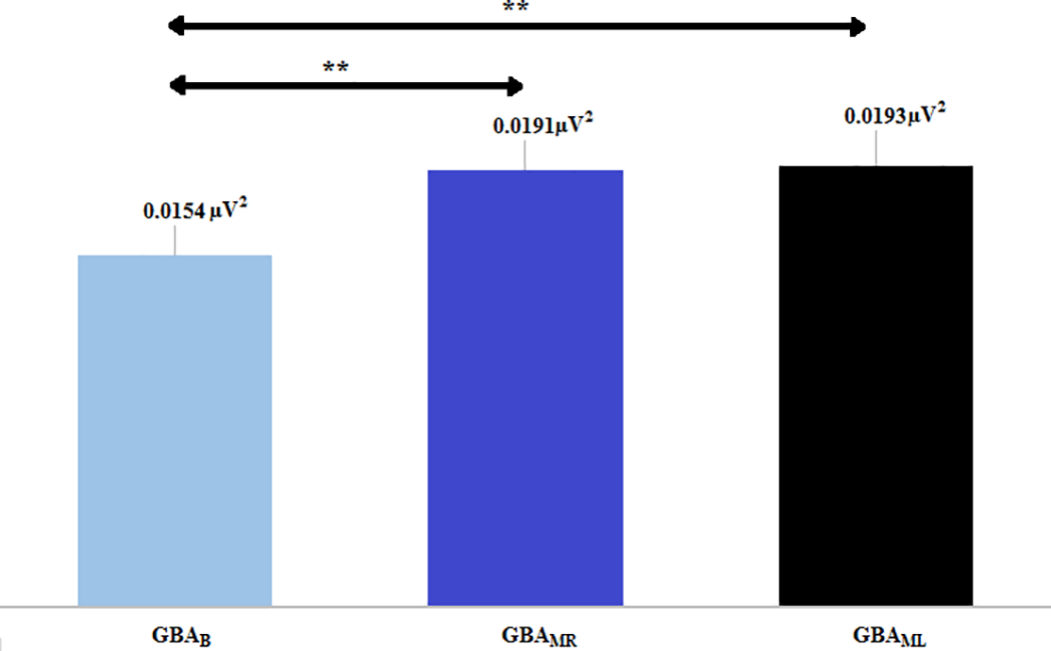
Average values of the GBA (PSD in μV^2^) at rest and during the movement of both hands. It is observed that GBA_MR_ > GBA_B_ and GBA_ML_ > GBA_B_. There are no significant differences between the movements of both hands (GBA_MR_ ≈ GBA_ML_). GBA = Gamma Band Activity, B = Basal, MR = Motor Right, ML = Motor Left. Asterisks indicate highly statistically significant difference (p<0.01).

Fig 2 shows the GBA values of the baseline part of the experiment (GBA_B_) were compared with the ones from the motor part of the experiment (GBA_MR_ and GBA_ML_). We also compared values of the right (GBA_MR_) and left (GBA_ML_) hand. The average values (16 subjects) of GBA are the following (Mean (CI 95%)): GBA_B_ = 0.0154 (0.0119–0.0189), GBA_MR_ = 0.0191 (0.0144–0.0237) and GBA_ML_ = 0.0193 (0.0141–0.0243). There was a significant increase (p < 0.001) of the GBA of both hands in movement with respect to the GBA at rest (GBA_MR_ > GBA_B_ and GBA_ML_ > GBA_B_). No significant differences were found between both hands (GBA_MR_ ≈ GBA_ML,_ p = 0.799).

### Second step: Obtain the ERS values

The average values (16 subjects) for right and left hand of the ERS are the following (Mean (CI 95%)): ERS_R_ = 1.2365 (1.1467–1.3161) and ERS_L_ = 1.2486 (1.1402–1.3212).

The increase (ERS) of the motor GBA over the basal GBA expressed as % is:

$${ERS}_{R(\%)}=\frac{{GBA}_{MR}}{{GBA}_{B}}=23.65\%$$

$${ERS}_{L(\%)}=\frac{{GBA}_{ML}}{{GBA}_{B}}=24.86\%$$

There was no significant difference between ERS_R_ and ERS_L_.

### Third step: Prove that there is no correlation between the EMG amplitude and the GBA during movement

Table 2 provides the average values of the EMG amplitude expressed as EMG_PAR_ and the GBA during movement (GBA_MR_, GBA_ML_), for both hands.

**Table 2:** EMG_PAR_ and GBA values (average and CI 95%).

	Left hand	Right hand
**EMG**_**PAR**_ **(μV/μV)**	**11.8** (10.9–12.8)	**12.3** (10.5–14.1)
**GBA (μV**^**2**^**)**	GBA_ML_ =	GBA_MR_ =
	**0.0191** (0.0144–0.0237)	**0.0193** (0.0141–0.0243)

EMG = Electromyogram, GBA = Gamma Band Activity, ML = Motor Left, MR = Motor Right.

There were no significant correlations between both parameters in both hands, right (EMG_PAR right_, GBA_MR_; r = -0.043, p = 0.874) and left (EMG_PAR left_, GBA_ML_; r = 0.271, p = 0.310), indicating that the GBA obtained during the motor task was independent from the amplitude (EMG) of the performed movement.

### Fourth step: Calculation of the GBA for the three intervals of the motor task and calculation of the index of plasticity

Here we expected to prove that the GBA_B_ did not increase over time while the GBA_MR_ and GBA_ML_ increased with movement repetition (training). In order to do that, the average values of the GBA were calculated for each of the intervals of the experiment (initial, middle and final) which were previously defined. Baseline activity was also divided into intervals equivalent to the ones of the motor task in order to compare them. The average values (16 subjects) of GBA of each interval are shown in Table 3.

**Table 3:** GBA values: Basal, right and left in the different intervals of the experiment.

	Interval		
	Initial	Middle	Final
**GBA**_**B**_ **(μV**^**2**^**)**	0.0152	0.0156	0.0155
**GBA**_**MR**_ **(μV**^**2**^**)**	0.0179	0.0195	0.0203
**GBA**_**ML**_ **(μV**^**2**^**)**	0.0172	0.0195	0.0215

Total sample (16 subjects). GBA = Gamma Band Activity

MR = Motor Right, ML = Motor Left.

There was a significant increase of the final activity compared to the initial activity for the motor task (GBA_MR_FIN_ > GBA_MR_INI_ (p = 0.044) and GBA_ML_FIN_ > GBA_ML_INI_ (p = 0.042)) while there was no significant difference for the baseline activity (GBA_B_FIN_ > GBA_B_INI_, not significant, p = 0.086).

**Fig 3:**
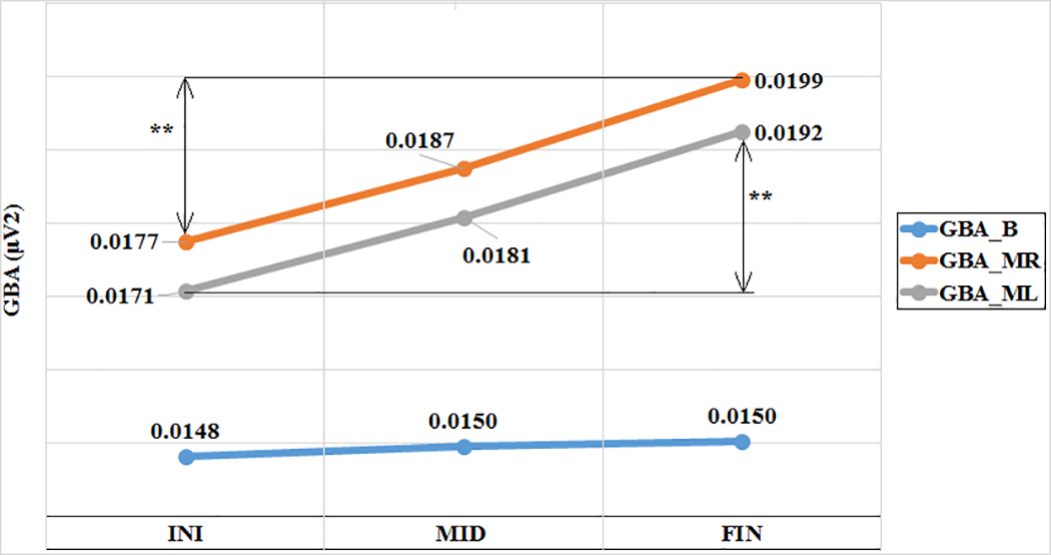
Average values of the GBA (PSD in μV^2^) at rest and during the movement of both hands. The values are represented according to the time intervals (initial, middle, final) for the right-handed subjects (n = 11). GBA = Gamma Band Activity, B = Basal, MR = Motor Right, ML = Motor Left, INI = Initial, MID = Middle, FIN = Final. Asterisks indicate statistically significant difference.

Fig 3 shows the average values of the GBA were also calculated by intervals only for right-handed subjects (n = 11: 7 males, 4 females). We did not calculate that for ambidextrous (n = 3) or for left-handed subjects (n = 2) due to the limited sample.

Based on the GBA values by intervals, the indices of plasticity were calculated for the movements of both hands (IP_R_, IP_L_) and the variation of the final baseline activity with respect to the initial one (ΔGBA_B_). The variation of the baseline activity is considered only to demonstrate that it does not undergo significant changes over time and to compare it to the plasticity indices (IP_R_, IP_L_).

Table 4 shows the plasticity indices for the total sample (n = 16) and for the sample of right-handed subjects (n = 11). Significant differences were found between the ΔGBA_B_ and both plasticity index (ΔGBA_B_ vs. IP_R,_ p = 0.04 and ΔGBA_B_ vs. IP_L,_ p = 0.03). No significant differences were found between both plasticity index (IP_R_ vs. IP_L_, p = 0.77).

**Table 4:** IP_R_, IP_I_ and ΔGBA_B_ values for the total sample and for the right-handed subjects.

	Total sample (n = 16)	Right-handed (n = 11)
**ΔGBA**_**B**_	1.0168	1.0176
**IP**_**R**_	1.1144	1.1075
**IP**_**L**_	1.2442	1.1218

GBA = Gamma Band Activity, IP = Index of Plasticity, R = Right, L = Left.

### Fifth step: Test the coherence of the results with the neurophysiology

**Fig 4:**
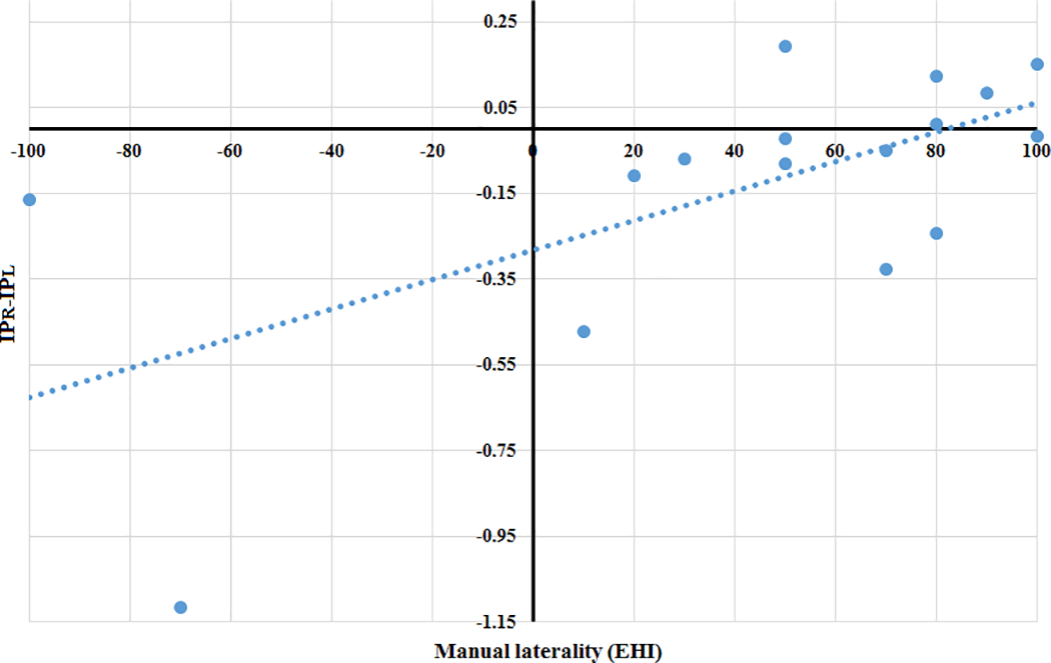
Correlation between the manual laterality (Edinburgh Handedness Inventory (EHI)) and the difference of indices (IP_R_−IP_L_). Total sample 16 subjects. IP = Index of plasticity, R = Right, L = Left.

Fig 4 shows the correlation between IP, determined by the difference of indices (IP_R_−IP_L_) and manual laterality (r = 0.624, p = 0.01). The regression line shows a significant relationship between manual laterality (EHI) and the indices difference (IP_R_−IP_L_).

## Discussion

The purpose of this study was to describe a method to quantify the effects of the voluntary movements (motor task) on the motor cortex through the quantification of increases of the GBA. The knowledge and the evaluation of these cortical plastic changes could be applied in the clinical practice.

We found GBA changes during repeated practice of a specific movement. There was a significant increase in GBA at the end of the task, compared to the initial GBA for the motor task (GBA_M_FIN_ > GBA_M_INI_) (p < 0.05). No differences were found at baseline (GBA_B_FIN_ ≈ GBA_B_INI_).

In previous experiments with magnetoencephalography (MEG), it has been verified that the same motor task used in this study, causes activation of the motor cortical network (primary and supplementary motor areas) with both voluntary and imagined movement [14,15].

Our findings suggest that evaluation of training-induced changes in GBA and IP can improve the assessment of brain activity. In this work, these changes have been related with voluntary movement.

In order to quantify training-related brain plastic changes, it would be necessary to quantify the changes in the activated cortical areas before and during an intervention. This cortical measure can be performed by means of different neuroimaging and neurophysiology techniques; EEG, MEG, positron emission tomography (PET), TMS and functional magnetic resonance imaging (fMRI) [1,3,6,7,16,17]. All of these techniques provide an approximate and indirect measure of the activated cerebral areas although the only real and direct measure of the activated cerebral areas (gamma activity) during movement would be electrocorticography (ECoG). The problem of ECoG is the high invasivity [18].

Studies performed with conventional EEG to quantify the effects of exercise on the brain are easier, but they only measure changes in the power spectrum of the different frequency bands of the EEG [19–21]. A more approximate measure with EEG would be the quantification of cortical motor activity with ERS, but it could only measure the activity increase during exercise in relation to baseline activity [8]. This measure would be more closely related to cortical excitability than with the size of the cortical area involved.

However, this experiment could indirectly measure plastic changes by comparing the gamma activity of the EEG at the beginning (GBA_M_INI_) with the gamma activity at the end of the repeated exercise (GBA_M_FIN_). Specifically, we defined an Index of Plasticity, $IP=\frac{{GBA}_{M\_FIN}}{{GBA}_{M\_INI}}$, which should be > 1 according to the initial hypothesis GBA_M_FIN_ > GBA_M_INI_. We could also define IP based on ERS:

If ${ERS}_{FIN}=\frac{{GBA}_{M\_FIN}}{{GBA}_{B}}$ and ${ERS}_{INI}=\frac{{GBA}_{M\_INI}}{{GBA}_{B}}$ then:

$$IP=\frac{{ERS}_{FIN}}{{ERS}_{INI}}=\frac{{GBA}_{M\_FIN}/{GBA}_{B}}{{GBA}_{M\_INI}/{GBA}_{B}}=\frac{{GBA}_{M\_FIN}\times {GBA}_{B}}{{GBA}_{B}\times {GBA}_{M\_INI}}=\frac{{GBA}_{M\_FIN}}{{GBA}_{M\_INI}}$$

Thus, the parameter IP described in this paper quantifies the activation of new areas and their progress over time with the repetition of the exercise, that is, plastic changes.

One important question is whether these training-induced GBA changes are related to a greater number of cortical motor areas involved in a specific motor task, in other words, if the proposed IP is indeed measuring plasticity.

In ECoG studies, the activated areas during movement have been compared with the increases of the GBA, demonstrating that ERS values change with the different motor tasks and with the different activated areas, consequently, it can be approximately assumed that for the same motor task, the greater the underlying area, the higher the value of ERS [8].

Therefore, the IP mathematically defined by changes of the ERS, would indicate an approximate measure of plasticity. Cortical area maps obtained with other techniques as TMS, fMRI, ECoG, etc.) could be carried out to confirm this fact.

To demonstrate that this experiment measure the proposed IP, we tested the initial hypotheses following different steps through the obtained results:

- The first step was to prove that the GBA during movement is significantly higher than the baseline GBA. Fig 2 shows a significant increase of the GBA with the movement with respect to baseline GBA (GBA_MR_ > GBA_B_ and GBA_ML_ > GBA_B_ (p < 0.01 in both hands)). This first step proved that the described task produced a quantifiable and significant GBA, what implies that the activation of the motor cortex can be measured.
- The second step consisted in calculating the ERS values: ERS_R_ = 23.65% and ERS_L_ = 24.86%. These ERS values were in the same range as the others reported in previous studies with motor tasks similar to the one used in the current study, which obtained ERS values ≈ 10–30% [10,22–24].
- In the third step we proved that there was no significant correlation between the movement intensity of the hand (EMG_PAR_) and the GBA_M_. This means that the activation of the motor cortex (GBA_M_) was measured independently from the amplitude of EMG (EMG_PAR_) obtained during the motor task. This result is in line with other similar experiment in which the amplitude, duration and frequency of oscillations were not related to movement parameters [25].
- The fourth step corresponded to the calculation of the IP. Fig 3 demonstrated that the GBA_B_ does not increase over time (GBA_B_FIN_ > GBA_B_INI_ (not significant)), while the GBA_MR_ and GBA_ML_ increase with the repetition of the movement (GBA_MR_FIN_ > GBA_MR_INI_ (p < 0.05) and GBA_ML_FIN_ > GBA_ML_INI_ (p < 0.05)).
- The fifth step provides evidence for the coherence of results with neurophysiology. As shown in Table 4, we observed an IP_R_ and IP_L_ asymmetry (not significant) in the total sample (n = 16), possibly because the sample is heterogeneous. However, if only the right-handed subjects (n = 11) were considering, IP_R_ and IP_L_ values were similar.
  The relation of the IP with manual laterality could be established; the (Pearson) correlation between manual laterality (EHI) and the indices difference (IP_R_−IP_L_) was positive (r = 0.624) and significant (p = 0.01) (Fig 4). Despite the negative value (IP_R_−IP_L_) presented by some of the right-handed subjects, the general trend showed by the regression line is coherent with the neurophysiology because the more right-handed the subject is, more brain cortex recruits when the right motor task progresses, and vice versa. The more left-handed the subject is, the more recruitment of brain cortex is observed as the left motor task progresses.

Based on the 5 steps stated above, we proved our study hypotheses. Due to plastic changes, GBA power is significantly higher at the end of a motor task than at the beginning. This method obtains GBA by means of the EEG and defines the IP; our results are significant and coherent with neurophysiology.

The methodology we describe here is an objective measure of the GBA that could be used as an approximate and indirect measure of the neuronal plasticity. Other methods based on neuroimaging and neurophysiology like the MEG, PET, TMS and fMRI could be more direct, but they are not as accessible due to their complexity and high cost, and they would not be very practical in sports medicine or in routine clinical practice.

Our study has limitations. The plastic capacity of the brain could be measure indirectly with the method presented in this paper. It is not possible to establish a direct comparison with other previous studies because there are not publications which describe a measure of brain plasticity similar to the IP described here. In this study we evaluated GBA changes induced by exercise which implies activation changes of different cerebral areas, while in previous research described functional changes (MEG, FMRI, TMS, and PET) [6,7,16,17] or focused only on the power spectrum of the EEG [19–21]. As a second limitation, the sample that we used was very small and it was not homogeneously distributed in manual laterality (11 right-handed, 2 left-handed and 3 ambidextrous subjects). It would be necessary to introduce more subjects and balance the number of left-handed and ambidextrous subjects. Regarding the method, it could be improved and simplified in further studies taking into account the following:

- Conduct the experiment with closed eyes, using an auditory stimulus or making a constant movement of the hands without cue, in order to avoid the frequent blinking artifact.
- Make the test more comfortable for the subject, reducing the total recording time (shorting the time of each trial).

Finally, our study did not evaluate longitudinal changes beyond what was found in a brief session of training. Future studies should evaluate changes in IP over longer periods of time and in larger sample sizes. Other interesting cases to study are to compare results between gender (male/females) or between different age groups (young/old).

## Conclusions

In this study we describe a method that could evaluate training-related plastic changes. The described method is applicable on a daily basis by using a simple task using the EEG which is a low cost technique and widely available. A simple numeric parameter, the IP, is proposed, which could indirectly but objectively measure the changes caused by the physical training in each individual.

This method could be used as a future diagnostic test in the follow-up of patients undergoing rehabilitation, assessing the recovery of their neurological disability after a stroke or brain injury. It could also have potential applications in the fields of sports medicine.

## Supporting information

S1 Table Parameters value of each subject of the sample. EHI = Edinburgh Handedness Inventory, GBA = Gamma Band Activity, B = Basal, MR = Motor Right, ML = Motor Left, ERS = Event Related Synchronization, INI = Initial, MED = Middle, FIN = Final, ΔGBAB = variation of the basal activity, IP = Index of plasticity, EMG = Electromyogram, PAR = Peak Average Ratio.
(XLSX)
